# The antiproliferative potential and mechanism of action of metformin in MCF-7 cells

**DOI:** 10.2144/fsoa-2022-0080

**Published:** 2023-04-21

**Authors:** Zhibek Bekezhankyzy, Sholpan Nurzhan, Nurken Berdigaliyev, Shynggys Sergazy, Tilektes Maulenkul, Mohamad Aljofan

**Affiliations:** 1 Department of Biomedical Sciences, School of Medicine, Nazarbayaev University, Astana, 010000, Kazakhstan; 2 National Laboratory Astana, Nazarbayev University, Astana, 010000, Kazakhstan; 3 Khoja Akhmet Yassawi International Kazakh-Turkish University, Turkistan, 161211, Turkistan, Kazakhstan

**Keywords:** antiproliferative, apoptosis, autophagy, breast cancer, metformin

## Abstract

**Aim:**

The current study aimed to investigate the potential antiproliferative activity of metformin, the effective concentration range, and the mechanism of action.

**Materials & methods:**

Human breast cancer cells, MCF-7 were treated with a serial dilution of metformin (10–150 μM) for 24 and 48 h. Potential antiproliferative activity of metformin and its ability in inducing cellular apoptosis and autophagy were also investigated.

**Results:**

Metformin inhibited MCF-7 proliferation in a concentration and time dependent manner, with 80 μM as the most effective concentration. Compared with nontreated cells, metformin induced significant levels of autophagy and apoptosis, which were confirmed by the reduction of mTOR and BCL-2 protein expression.

**Conclusion:**

The study confirms the antiproliferative activity of metformin, which may likely occur through AMPK signaling pathway.

## Plain language summary

The antidiabetic drug, metformin is tested in this work for its possible ability to inhibit the growth of breast cancer cells. Using different concentrations of the drug over different time points, the results showed that the drug was able to inhibit cancer growth through different mechanisms. The results also showed that the drug inhibits cancer growth by stimulating program cell death (apoptosis), as well as autophagy, where the cell breaks old and abnormal cellular substances.

Metformin, (1,1-dimethylbiguanide hydrochloride) the first-line therapy for the treatment of Type 2 diabetes mellitus, has been reported to significantly reduce the risk of cancer [1]. A pilot case–control study in 2005, suggested that Type 2 diabetes mellitus patients who had used metformin had a lower risk of cancer [2]. Other observational studies reported an increase in survival rate of cancer patients who used metformin [3–5]. An epidemiological study reported a lower cancer specific mortality among subjects with diabetes using metformin compared with diabetics on other treatments [6]. Patients with uncontrolled diabetes mellitus have 20–25% risk of breast cancer, which is the most common cause of cancer morbidity among women between the ages of 40 and 70 years old [7].

These reports are further supported by several preclinical studies that reported anticancer potential of metformin against several types of cancer in humans, including prostate, lung, breast and pancreas cancers [8]. In animal studies, metformin delayed the onset of mammary cancer and other tumors in tumor prone mice [5,9].

The primary site of action of metformin is in the liver where it activates adenosine-mono-phosphate activated protein kinase (AMPK) and suppresses hepatic glucose production via the suppression of cAMP response element-binding protein (CREB) mediated gluconeogenic gene activation [10]. However, the undetermined antiproliferative mechanism of action is thought to be related to its glucose lowering ability and activation of the AMPK, which lowers glucose levels, inhibits cell growth and suppress tumor growth [11].

Other important factors that may influence its antiproliferative ability includes concentration and treatment time. For example, some metformin studies reported potential antiproliferative activity only with high concentrations (mM range), which do not reflect the clinical actions of the drug as it is used for the treatment of Type 2 diabetes [12]. At a concentration of 2 mM, metformin inhibited glucose starvation induced endoplasmic stress and autophagy in endothelial cells [13]. To the contrary, several studies reported significant antiproliferative activities at various micromolar concentrations including Marinello *et al.*, which reported that 30 uM of metformin treatment was enough to significantly reduce cellular proliferation of human breast cancer cell lines (MDA-MB-231) [14]. Also, Liu and colleagues reported that a combination of 10 μM of metformin and ionizing radiation resulted in approximately 80% decrease of hepatoma cell viability compared with ionizing radiation alone [15].

Therefore, it is very important to determine the mechanism of action, the effective antiproliferative concentration and the suitable treatment time of metformin. Confirming these factors will improve the understanding of the antiproliferative activity of metformin and its suitability as a potential antiproliferative drug.

## Materials & methods

### Metformin preparation

Metformin hydrochloride (1,1-Dimethylbiguanide hydrochloride 97%, Sigma-Aldrich, Germany) was dissolved in distilled water for preparation of a stock solution with 300 μM concentration. To avoid any impurities or undissolved particles, the stock solution was filtered by a syringe filter with PES membrane 0.22 μm.

### Cellular proliferation assay

Cellular proliferation was detected using the MTT (3-[4,5-dimethylthiazol-2-yl]-2,5-diphenyltetrazolium bromide) method following the methodology of the manufacturer (Cell Proliferation Kit I [MTT], Roche, was purchased from Sigma Aldrich and stored at -20°C). Breast cancer cells MCF-7 (HTB-22™ sourced from ATCC, Germany) were cultivated in normal glucose Dulbecco’s modified Eagle’s medium ([DMEM], Sigma, MO, USA) supplemented with fetal bovine serum ([FBS]; 10% [v/v]; Sigma) and streptomycin and gentamycin (1% [v/v]; Sigma). Cells were seeded at 10,000 cells/100 μl in a 96-well plate and incubated for 20 h at 37°C and 5% CO_2_ (Binder CO_2_ incubator, Model CB-S 170). On the next day with a final volume of 100 μl, serial log dilutions of metformin (concentration from 9 to 150 μg/ml) and positive control (5% DMSO) was performed. MCF-7 cell lines were incubated in the presence of metformin and 5% DMSO at 37°C and 5% CO_2_ for a 24 and 48 h timelines. Cells were treated with MTT and incubated for 4 h and then formazan crystals were dissolved by addition of DMSO. Absorbance was measured using a 96-well imaging reader (iMark Microplate Reader, Bio-Rad, Inc., USA) at 570 nm. Antiproliferative activity (%) was calculated by dividing the treated reading over the control reading. Cell viability (%) was calculated by formula:

$$\left[\frac{{\text{Treated}}_{570\;\text{nm}}}{{\text{Control}}_{570\;\text{nm}}}\right]\times 100$$

### Autophagy assay

Cellular autophagy was performed using the Autophagy Assay kit (Abcam, Cambridge, UK) following the manufacturer’s guidelines. MCF-7 cells were cultured in black 96-well plates with 10,000 cells/ well density and then treated with metformin in different concentrations, rapamycin and galangin as controls and incubated for 24 h at 5% CO_2_ and 37°C. After the incubation, growth medium was discarded and 100 μl of autophagosome detection reagent working solution was added to wells (sample and control) and incubated for 30 min at 5% CO_2_ and 37°C conditions. Wells with cells were washed three-times with a wash buffer (100 μl) and the buffer was removed gently from the wells, not disrupting the cells. Fluorescence intensity was measured using an imaging reader (Cytation ™ 5, Bio-Tek Instruments, Inc., VT, USA) at λ_ex360_/λ_em520_ nm.

### Apoptosis assay

Apoptosis was performed using the Muse Annexin V and Dead cell kit - 100 tests (part number MCH100105) following the manufacturer’s guidelines. MCF-7 cells were cultured in a 6-well plate with 400,000 cells/well density and then on the next day were treated with 85 μM metformin and 3% DMSO as positive control, and incubated for 24 h at 5% CO_2_ and 37°C. After the incubation floating and adherent cells were collected in a 15-ml tube. During preparation of adherent cells for apoptosis assay, they were trypsinized and then trypsin was removed by centrifugation at 300 g RCF (relative centrifugal force) for 5 min and the cell pellet was resuspended in 1 ml of medium. Cells were counted using hemocytometer and nontreated cells were used as negative control and cells treated with DMSO were used as positive control. 100 μl of cell suspension was mixed with 100 μl of Muse Annexin V and Dead Cell reagent, vortexed and incubated for 20 min at room temperature in the dark. Before acquisition cells were resuspended and then loaded to the Guava Muse Cell Analyzer.

### Protein expression using western blot

MCF-7 cell lines were grown for 20 h and then treated with 85 μm of metformin for 24 and 48 h. About 100 μl of samples were prepared by diluting proteins in Laemmli Buffer until the 10 μg/20 μl concentration with addition of 14 μl of 70% glycerol and 4 μl of bromophenol/mercaptoethanol. Before loading into the wells samples were heated at 95°C for 5 min and vortexed. Protein concentrations were determined with the help of BCA assay (71285-3, Novagen) and 10 μg of each sample were used for the protein expression using western blot. Protein transfer was performed by wet transfer method where gel is immersed in 1x Transfer Buffer and sandwiched between two pieces of filter paper. The order of sandwich was sponge/filter paper/gel/PVDF membrane/filter paper/sponge that were clamped tightly avoiding bubbles between gel and membrane. PVDF membrane was activated by 96% ethanol before preparing a gel/membrane sandwich. Sandwich was placed between positive and negative electrodes submerged in 1x Transfer Buffer and 100V electrical field was applied for 1 h. Due to high voltage, the ice block was submerged into the transfer buffer to avoid overheating of the buffer. Blocking solution (5 g of non fat dried milk dissolved in 100 ml of TBST) was added to the membranes and left for 1 h on the shaker to block nonspecific bindings. Membranes were incubated overnight with primary antibodies specific to alpha-tubulin, to mammalian target of rapamycin (mTOR) and B-cell lymphoma 2 (BCL-2) in the cold room at 4°C. After washing the membrane with TBST, a secondary antibodies: horseradish peroxidase conjugated secondary antibodies anti rabbit and anti goat was added and incubated for 1 h at room temperature. For the band detection enhanced chemiluminescence (ECL) (Amersham™, ECL™ start western blotting detection reagent) solution was prepared by mixing Solution A and Solution B (1:1 ratio) and added drop by drop on the surface of the membrane (100 μl/membrane) and incubated for 90 s before the visualization. Detection and quantification of the bands were performed using an NIH ImageJ software system.

### Statistical analysis

Data were analyzed as mean ± standard deviation unless otherwise reported. Statistical significance was demonstrated by a priori level of p < 0.05 and significance by chi-square test. Each point on the graph demonstrates an independent experiment. Data collection and analysis was performed on a Microsoft Office Excel^®^ (2013) spreadsheet. Statistical tests were performed using GraphPad Prism version 6.00 software (GraphPad Software, CA, USA).

## Results

### Determination of effective concentration range

**Figure 1.**
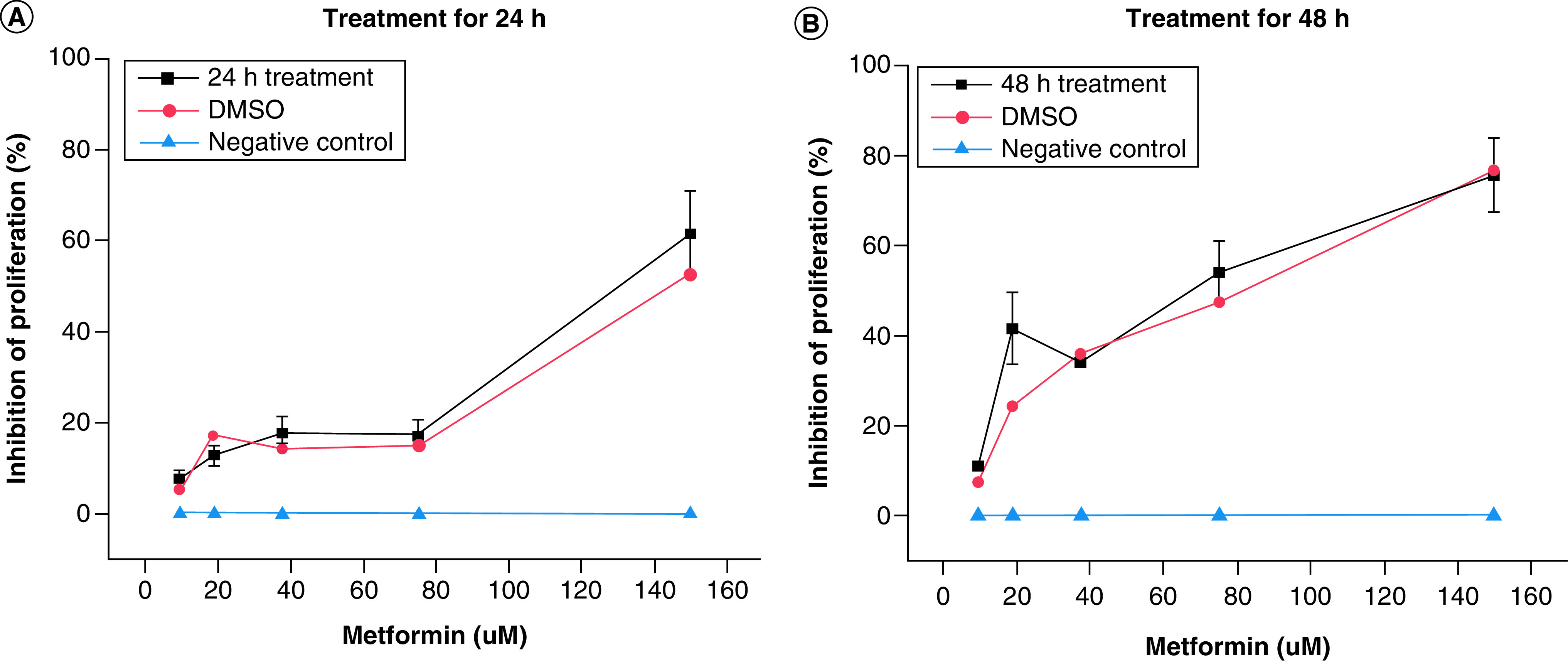
Determination of effective concentration range. Shows the antiproliferative potential of metformin expressed as percentage of inhibition of cellular proliferation versus drug concentration. Cells treated with serial dilutions of metformin (10–150 μM) for **(A)** 24 h and **(B)** 48 h . Nontreated cells were used as a negative control (blue line) and serial dilution of DMSO (1–0.06%) was used as positive control (red line). Data are expressed as mean ± SD (n = 6 for each presented value). SD: Standard deviation.

To determine the antiproliferative concertation range and optimal treatment time of metformin, cells were treated with a serial dilution of metformin (10–150 μM) for 24 and 48 h separately. A serial dilution of DMSO (1–0.06%) were used as a positive control, and nontreated cells as a negative control. At 24 h metformin treatment, there was a slow increase in inhibition of cellular proliferation with concentrations less than 70 μM, but starting at 75 μM and above, there was significant increase in the inhibition of cellular proliferation compared with negative control with a potency comparable to that of the positive control (Figure 1A). However, expect for 10 μM, all of the concentrations of metformin at 48 h significantly inhibited cellular proliferation more than the 24 h treatments and at 80 μM of metformin showed significantly more cellular inhibition than the positive control and that at highest concentration showed similar potency to 1% DMSO (Figure 1B). Therefore, 80 μM concentration was selected for the rest of the experiments, as it was shown to be the most effective concentration of metformin in both timeframes (24 and 48 h).

### Metformin effect on autophagy

**Figure 2.**
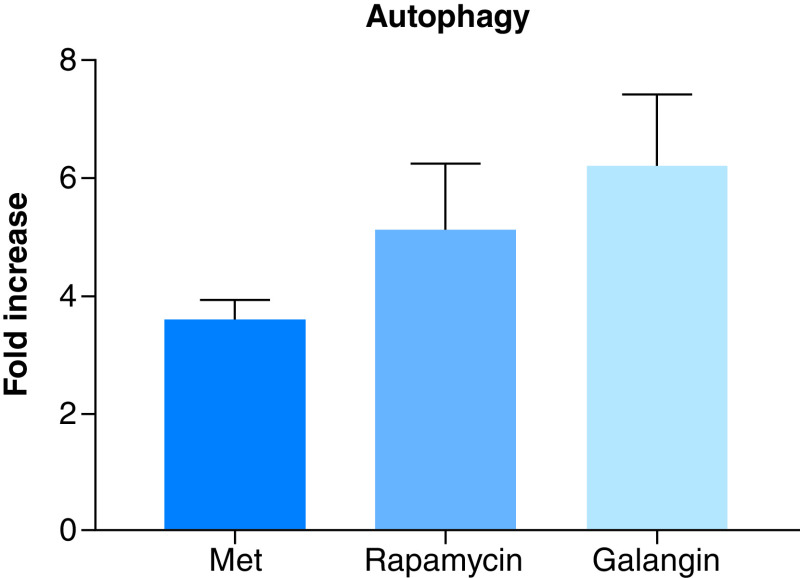
Metformin effect on autophagy. Shows the autophagy induction ability of metformin compared with rapamycin and galangin on MCF-7 as measured by fluorescence absorbance, which reflects autophagocytosed cells. Cells treated with the selected effective concentration of 80 uM of metformin for 24 h, produced less than fourfold increase in autophagy compared with negative control. Whereas galangin and rapamycin, the two positive controls, induced sevenfold and fivefold increase in autophagy, respectively. Data are expressed as mean ± SD; n = 6. SD: Standard deviation.

One of the possible antiproliferative mechanism of action of metformin is autophagy. Thus, the autophagic potential of metformin was determined using a commercially available kit, which measures the number of autophagocytosed cells. At a concentration of 80 μM, metformin induced an approximately fourfold increase in the level of autophagy relative to negative control (Figure 2). While the observed autophagic levels were less than the two used positive controls, rapamycin and galangin, it can safely be assumed that metformin is an inducer of autophagy in MCF-7 cells and that it may have possibly contributed to its antiproliferative activities.

### Effect of metformin on apoptosis

**Figure 3.**
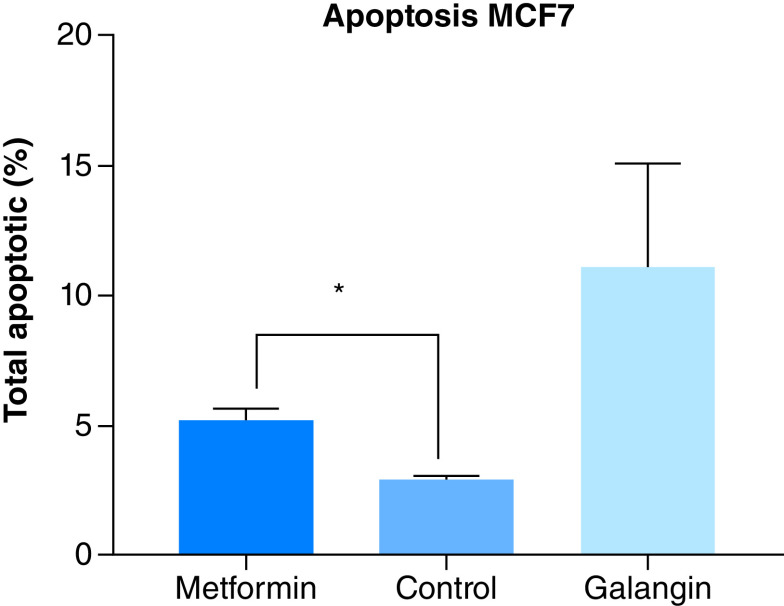
Effect of metformin on apoptosis. The apoptotic activity of metformin compared with negative control (nontreated cells) and positive control (galangin). At 24 h incubation, treatment with 85 uM of metformin produced a significantly higher percentage of apoptosis compared with control (p < 0.01). Data are expressed as mean ± SD; n = 3. *Represent a significant difference. SD: Standard deviation.

The apoptotic potential of metformin was measured by a commercially available fluorescence Caspase 3/7 assay. Treatment with the positive control galangin, resulted in a significantly higher apoptotic levels than negative control (p < 0.0001) and metformin treatment (p < 0.001). However, compared with the negative control, treatment with 80 μM of metformin produced a significant level of apoptosis in MCF-7 cells (p < 0.01) (Figure 3).

### Determination of apoptotic protein expression

**Figure 4.**
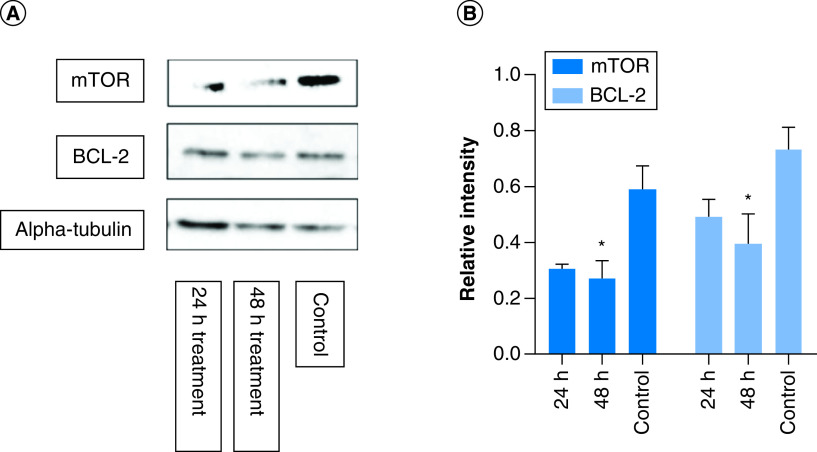
Determination of apoptotic protein expression. MCF-7 cells were treated with metformin separately for 24 and 48 h. The western blot images above show mTOR, BCL2 and alpha-tubulin protein expressions from each of the different time point. The graphs represent relative expression intensity of mTOR and BCL-2 in relation to alpha-tubulin from control MCF-7 cells (nontreated). Compared with control, metformin treatment for 24 h significantly reduced protein expression levels of mTOR (p = 0.03) and BCL2 expression (p = 0.04). However, at 48 h treatment, the protein expression of mTOR and BCL2 were further reduced (p = 0.008 and p = 0.01, respectively). The samples derive from the same experiment and that gels/blots were processed in parallel. Data are expressed as mean ± SD; n = 3. *Represent a significant difference. SD: Standard deviation.

mTOR is a protein kinase regulating cell proliferation and BCL-2 is an antiapoptotic protein, both of which have been identified as potential targets for antiproliferative activity of metformin (Aljofan & Riethmacher, 2019). Therefore, the effect of metformin treatment and the treatment time on the expression level of these proteins were investigated using western blot analysis. The color intensity of the protein band from the western blot corresponds to protein expression level, quantified using Image Analyzer Software and the protein expression determined by calculating the ratio of mTOR and BCL-2 to that of alpha tubulin, the housekeeping protein. In comparison to control, metformin treatment significantly reduced the protein expression of mTOR (p = 0.03) and BCL-2 (p = 0.04) in a time–dependent manner; thus, confirming the apoptotic potential of metformin (Figure 4).

## Discussion

This study investigated the concentration range and treatment time of metformin that produce antiproliferative activity against human breast cancer cells (MCF-7) as well as investigating possible mechanism of action. The results showed that metformin was able to inhibit MCF-7 cellular proliferation in a time and concentration manner. Expect the lowest concentration of 10 μM, at 24 h metformin significantly inhibited cellular proliferation in all used concentrations compared with negative control and more than the positive control at the highest concentration of 150 μM, with a further increase in the inhibition at 48 h.

The results showed that metformin was able to produce a significant level of autophagy, a homeostatic and evolutionarily conserved process involved in the removal of damaged or long-lived proteins and organelles [16]. Albeit not as high as the positive control, autophagy induction in MCF-7, could possibly be the mechanism of action or a significant contributor to the antiproliferative potential of metformin, which was previously shown to be one of the effective anticancer preventative and treatment pathways [17]. Chemotherapeutic drugs appear to trigger autophagy, in response to radiation stress and serum starvation [18].

Furthermore, animal studies showed that a defect in cellular autophagy is strongly associated with tumorigenesis [19] and that it could also stimulate tumor antigen cross-presentation improving tumor immune responses [20].

Furthermore, mTOR is a protein kinase involved in the regulation of protein synthesis and cell growth and a presumed target for metformin [21], was significantly reduced in response to metformin treatment both in 24 and 48 h. This finding further supports the involvement of autophagy as a major contributor to the antiproliferative activity of metformin. Autophagy is regulated by class I phosphoinositide-3-kinase (PI3K) and AMPK in response to mTOR signaling [22,23]. Thus, activation of AMPK inhibits mTOR activity and regulates autophagy [24,25].

However, as a stress response, autophagy may prevent cancer cell death via suppressing cellular apoptosis, or triggers apoptosis by activating caspase-3 pathway [18]. Autophagy could potentially induce protective responses that could inhibit cellular apoptosis and enable cancer cell adaptation to stressful environment. Activation of TFE3, a member of the MiTF/TFE family of transcription factors [26] that regulate energy metabolism and cancer survival may also induce protective autophagy [27]. An *in vitro* study by Tan *et al.* reported that metformin increased TFE3 reporter activity that resulted in autophagy in MCF7 cells [26]. Interestingly, the study reported that blocking of autophagy; increased metformin induced apoptosis, and hence improved anticancer activity of metformin. While this study supports the theory of autophagic protection against cancer treatment, there needs to be more studies in this area to further understand the relationship between metformin treatment and protective autophagy.

Interestingly, the mTOR signaling pathway also regulates apoptosis, programed cell death involved in various physiological and pathophysiological processes including cancer [28]. In normal cells, mTOR regulates cell growth and division, but in cancer cells, mTOR signals tumor growth, metastasis and invasion of healthy tissues [29,30]. Often it is activated in tumors to regulate gene transcription, protein synthesis and cell proliferation, differentiation of immune cells and regulation of tumor metabolism [30]. mTOR is tightly modulated by various factors including, pro- and antiapoptotic proteins of the Bcl-2 family, which regulates apoptosis by controlling mitochondrial permeability [31,32]. An increase in mTOR signaling was shown to promote tumor growth and progression, making it a viable anticancer target possibly used in the anticancer activity of metformin. This theory is confirmed in the current study that showed a time-dependent reduction in protein expression of mTOR and BCL-2 following metformin treatment. A number of previous studies reported anticancer treatment of metformin to likely be achieved by influencing mTOR signaling, including Amin *et al.* which suggested that metformin treatment reversed hamartomas through its effect on mTOR [27]. While this represent a potential antiproliferative mechanism of metformin, it certainly not the only mechanism.

## Conclusion

The results showed that treatment of MCF-7 cells with metformin inhibited cellular proliferation in a dose and time dependent manners through increasing autophagy and cellular apoptosis, which were further confirmed by the decrease in mTOR and BCL-2 proteins expression. Since AMPK regulates autophagy and apoptosis, it is therefore, safe to assume that the antiproliferative activity of metformin is achieved via an AMPK signaling pathway. Thus, the current results warrant further investigation to determine the exact antiproliferative mechanism of metformin.

**Summary points**

- This work invested the antiproliferation potential of metformin and the possible mechanism of action.
- Different concentrations of the drug was added to breast cancer cells, MCF7, over different time points.
- The results showed significant differences in the cellular proliferation.
- Cellular proliferation was inhibited in time and concentration manner.
- Several techniques used to determine the antiproliferative mechanism of the drug, showed possible involvement of apoptosis and autophagy.
- The relationship between autophagy and apoptosis in anticancer is complex and more studies are needed to illustrate the effect in anticancer.
